# 3D bioprinting for orthopaedic applications: Current advances, challenges and regulatory considerations

**DOI:** 10.1016/j.bprint.2020.e00103

**Published:** 2020-12

**Authors:** D. Stanco, P. Urbán, S. Tirendi, G. Ciardelli, J. Barrero

**Affiliations:** a European Commission, Joint Research Centre (JRC), Ispra, Italy; b Department of Mechanical and Aerospace Engineering, Politecnico di Torino, Corso Duca degli Abruzzi 24, 10129, Turin, Italy

**Keywords:** 3D bioprinting technology, Tissue engineering, Bone, Cartilage, Regulatory framework, Standardization

## Abstract

In the era of personalised medicine, novel therapeutic approaches raise increasing hopes to address currently unmet medical needs by developing patient-customised treatments. Three-dimensional (3D) bioprinting is rapidly evolving and has the potential to obtain personalised tissue constructs and overcome some limitations of standard tissue engineering approaches. Bioprinting could support a wide range of biomedical applications, such as drug testing, tissue repair or organ transplantation. There is a growing interest for 3D bioprinting in the orthopaedic field, with remarkable scientific and technical advances. However, the full exploitation of 3D bioprinting in medical applications still requires efforts to anticipate the upcoming challenges in translating bioprinted products from bench to bedside. In this review we summarised current trends, advances and challenges in the application of 3D bioprinting for bone and cartilage tissue engineering. Moreover, we provided a detailed analysis of the applicable regulations through the 3D bioprinting process and an overview of available standards covering bioprinting and additive manufacturing.

## Abbreviations

MSCs: Mesenchymal stem cells
IPSCs: Induced pluripotent stem cells
ECM: Extracellular matrix
dECM: Decellularized extracellular matrix
CT: Computed tomography
MRI: Magnetic resonance imaging
PLGA: Poly(lactic-co-glycolic acid)
PCL: Poly-caprolactone
CaP: Calcium phosphate
TCP: Tri-calcium phosphate
HA: Hydroxyapatite
LAP: Laponite
GelMA: Gelatin-methacryloyl
PRP: Platelet rich plasma
BMP-2: Bone morphogenetic protein-2
VEGF: Vascular endothelial growth factors
ECs: Endothelial cells
CAM: Chorioallantoic
ITOP: Integrated tissue organ printer
ACI: Autologous chondrocyte implantation
ASCs: Adipose derived mesenchymal stem cells
BMSCs: Bone marrow-derived mesenchymal stem cells
VEGF: Vascular endothelial growth factor
HUVECs: Human umbilical vein endothelial cells
CB(6): Cucurbit[6]uril
DAH-HA: 1,6-diaminohexane-conjugated hyaluronic acid
hTMSCs: Human nasal inferior turbinate tissue-derived mesenchymal stromal cells
TGFβ: Transforming growth factor β
hAFMSCs: Human amniotic fluid-derived mesenchymal stem cells
OCP: Peripheral octacalcium phosphate
ICMRA: Coalition of Medicines Regulatory Authorities
EMA: European Medicines Agency
ATMP: Advanced Therapy Medicinal Product
MDR: Medical devices regulation
CAT: Committee for Advanced Therapies
GMP: Good manufacturing practice
ICH: International Council of Harmonisation standards
ISO: International Standardisation Organisation
ASTM: American Society for Testing and Materials

## 1 Introduction

Advances in regenerative medicine have introduced new and exciting opportunities for the development of patient-specific medical treatments. Tissue engineering, a subfield of regenerative medicine, combines the principles of biology, material sciences, engineering and medicine to obtain functional constructs that restore, maintain, or improve damaged tissues or whole organs [1].

Tissue engineering has the potential to revolutionise healthcare by providing artificially developed tissues and organs, which can be patient-specific in some cases, to overcome the limited efficacy and availability of organ transplants [2]. In addition, tissue engineering has shown promise in developing tools for improving methods for drug discovery and toxicity testing using *in vitro* tissue models [3].

Tissue engineering strategies are being investigated for a number of challenging musculoskeletal pathologies in the orthopaedic field. Several research efforts have focused on the generation of self-healing living tissue substitutes by combining patient-derived stem cells, *ad hoc* biomaterials and orthopedic surgery. However, although exciting developments have been reported in pre-clinical studies and clinical trials, tissue engineering still plays a relatively small role in patient treatment nowadays [4]. The poor batch-to-batch reproducibility of tissue engineered products, funding issues for product development, lack of knowledge of the regulatory requirements to get the approval for clinical trials and issues related to physician acceptance of a new treatment method limit the translation of tissue engineered constructs from bench to the clinic [5].

Bioprinting belongs to the family of additive manufacturing processes. The advantage of this biofabrication technique over conventional tissue engineering methods is the possibility of precisely combining cells, biomaterials and proteins into 3D living structures to recreate anatomical parts using medical images as blueprints. The development of 3D bioprinting is leading to a faster fabrication of tissues with increased complexity, resolution, and functionality, which can replicate more accurately tissue function *in vivo* [6].

Bioprinting provides a promising approach to tackle major obstacles in the generation of engineered living tissues and has been recently explored for different applications in regenerative medicine.

The use of 3D bioprinting has gained increasing attention in the last decade in the orthopaedic field, since cartilage injuries and bone loss have become increasingly prevalent in modern societies. 3D-printed customised prosthetics and implants are already available for patient-specific therapies, enabling bone, cartilage or osteochondral tissue regeneration.

In this review, we summarised the main advances in the production of 3D bioprinted bone and cartilage substitutes, focusing on the latest progress on the development of bioinks and optimisation of the bioprinting process. We also investigated the regulatory framework applicable for 3D bioprinted products and through the different steps of the biofabrication process. Finally, we reported on available standards in order to explore possible challenges for manufacturers and the healthcare system for the implementation of the bioprinting technology.

## 2 Search strategy

**Fig. 1:**
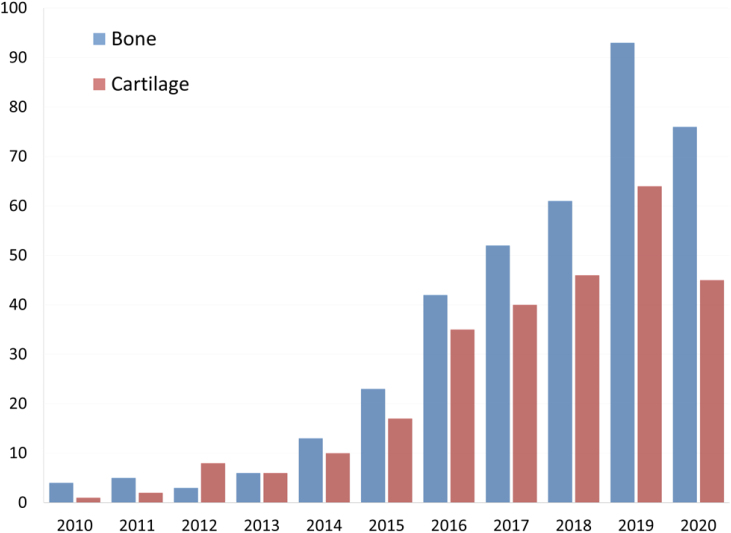
**Number of publications per year on bioprinting for bone and cartilage applications during the last decade.** Publications retrieved from PUBMED using the keywords described in the search strategy.

The impact of 3D bioprinting technology in orthopaedics was evaluated analysing peer-reviewed scientific articles in the field of bone and cartilage tissue engineering. A literature search was conducted to extract relevant articles related to 3D bioprinting applications in orthopaedics using PUBMED. The keywords used were: (“3D bioprinting” OR “bioprinting”) AND (“bone OR “cartilage”). The searches were performed until March 2020 and the language was limited to English. Since bioprinting is still in its initial stage of development, no restrictions to publication year were applied. Additional papers were identified through automated database notifications of new publications according to our search terms until June 2020. 654 publications were retrieved following our search strategy. The number of publications found for each year in the last decade is shown in Fig. 1. The increasing number of publications retrieved in the last years indicates the growing interest in this field.

## 3 Basic aspects of 3D bioprinting technology

Three-dimensional bioprinting is an additive manufacturing technology based on a layer-by-layer production of highly precise 3D structures. The controlled placement of biomaterials and living cells allows mimicking the morphology and functionality of the native target tissue with high reproducibility and repeatability. Bioprinted products possess a precisely controlled architecture such as external shape, internal pore geometry and interconnectivity and specific mechanical properties of the target tissue [7,8]. Providing an optimised microenvironment conducive to the growth of 3D structured tissue is an exciting prospect of bioprinting. The combination of a proper cell type and biomaterial to produce cell-laden 3D structures acting as “building blocks” for *in vivo* tissue formation is the main challenge of 3D bioprinting.

**Fig. 2:**
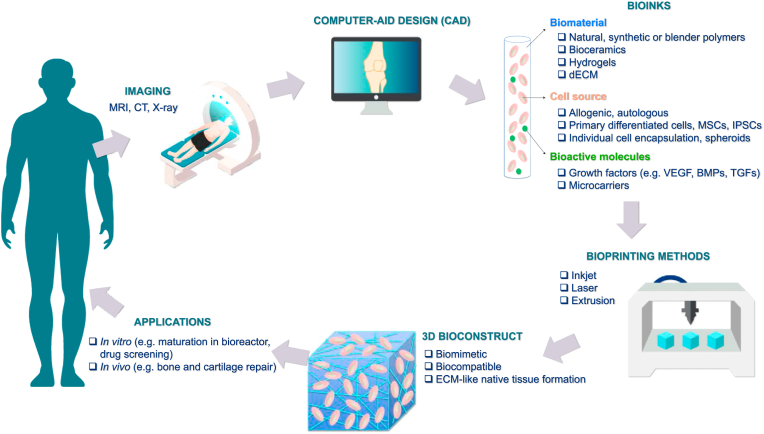
**3D bioprinting strategy.** The different steps for bioprinting 3D tissues are represented as a schematic illustration. The process starts with diagnostic images obtained using computed tomography (CT) and magnetic resonance imaging (MRI) scans from damaged tissues, which are used to obtain a computer-aid design. Common biomaterials, cell sources and bioactive molecules used in the bioinks are shown. These components have to be integrated using bioprinting techniques including inkjet, laser or extrusion-based methods. An appropriate biomaterial is crucial to obtain a final 3D bioconstruct resembling the microenvironment and growth cues of native tissues. A period of *in vitro* maturation in a bioreactor may be required before transplantation or use for *in vitro* applications.

Fig. 2 shows a schematic illustration of the bioprinting process, from medical imaging until the generation of 3D bioconstructs for different applications in tissue engineering. Bioink components include biomaterials, cells and bioactive molecules. Cell sources for bioprinting should closely mimic the physiological state of cells *in vivo,* since they are expected to maintain their *in vivo* functions under optimised conditions [7]. Cell sources may be allogeneic or autologous. Allogeneic cell sources are derived from unrelated donor tissues (such as bone marrow) and may be used to treat many patients whereas autologous cell sources are manufactured as a single lot from the patient being treated. In general, autologous cell sources are preferred to reduce risk of host immune system rejection. However, many primary cell types (such as chondrocytes or osteoblasts) are difficult to isolate and culture and their short lifespan is a limitation for the long-term functionality of any bioprinted constructs. For these reasons mesenchymal stem cells (MSCs), and more recently induced pluripotent stem cells (iPSCs), are largely employed. The presence of bioactive molecules such as growth factors could be employed to drive cell differentiation and proliferation. The different steps of the process and their components will be discussed below.

The bioink has the role of mimicking the tissue-specific extracellular matrix (ECM) components to offer an optimal environment for cell survival, proliferation and maturation. The bioink should provide the required strength and elasticity for mimicking the mechanical properties of the native tissue but at the same time enough stiffness to allow handling and implantation of the biomaterial [7]. In addition, tensile strength has also been identified as a key parameter for bioprintability. Specific mechanical properties values are required for bioprinting different tissues [9,10]. However, there is limited literature on the mechanical characterisation of the final 3D bioprinted product. Moreover, reproducing the exact tissue-like mineralisation, cellular diversity and mechanical properties of bone or cartilage tissue is not easily achievable and is still under investigation. Miramini et al. provide indicative material properties that are characteristic of articular cartilage and bone. In their review, values between <1 – 170 MPa were reported for elastic modulus in articular cartilage, whereas a range of 10–40 GPa was described for cortical bone. With regard to failure stress, Miramini et al. report a range of 4–50 MPa for articular cartilage and a range of 125–250 MPa for cortical bone [11].

Reproducing the tissue mineralisation, cellular diversity and mechanical properties of bone or cartilage is particularly challenging. On one hand, scaffolds with high mechanical properties are desirable for bone and cartilage tissue regeneration, but on the other some recent evidence reported greater cell differentiation and ECM production in a soft mechanical microenvironment [[12], [13], [14]].

Many parameters may influence the functionality and mechanical properties of the final bioprinted product. Different research groups are investigating the optimisation of printing parameters (e.g., pore size), cell density and initial mechanical properties of the biomaterials to obtain a 3D bioprinted construct resembling the mechanical properties of the native tissue.

For instance, Levett et al. showed that gelatin methacryloyl (GelMA)–hyaluronic acid (HA)–chondroitin sulfate (CS) scaffolds with low mechanical properties induced chondrogenic differentiation of the embedded chondrocytes and high production of cartilage ECM that resulted in significant increase in mechanical properties up to 150 kPa after 8 weeks of culture [13].

Common biomaterials for bioinks include synthetic or natural polymers, hydrogels and decellularized extracellular matrix (dECM) components. Hydrogels are often used as bioinks due to their biocompatibility and unique chemical and physical properties, such as high water content, nutrient and oxygen diffusion or *in vivo* biodegradability of the polymeric matrix. The hydrated nature of hydrogels resembles the water content present in many soft tissues like cartilage, contributing to the viscous and elastic behavior of the construct. Moreover, the majority of hydrogels possess specific cell-binding sites promoting cell attachment, spreading and differentiation. Both natural and synthetic polymers have been employed for the synthesis of hydrogels [9,15]. Natural polymers are derived from natural sources and have the notable property to resemble the native ECM cues. The use of natural ECM polymers as hydrogels for 3D bioprinting was pioneered by Pati and colleagues [16,17]. In their studies, decellularized extracellular matrices derived from cartilage, adipose and heart tissues were developed as temperature-sensitive hydrogels to encompass human MSCs and maintain their viability and differential lineage commitment into tissue-specific ECM formation. More recently, nanocellulose has been used due to its useful properties, such as natural origin, high water content, nanofibrillar composition highly resembling collagen fibril network, mechanical strength and shear thinning properties [[18], [19], [20]]. Synthetic polymers are fully synthesized in the laboratory with fine-tuned control of their composition and properties. The combination of natural with synthetic polymers allows modulating specific physico-chemical parameters of the hydrogel, such as elasticity, stiffness or thermal and conductive properties. Moreover, the polymer composition can be optimised to improve biodegradability and biocompatibility [9,10,15]. Thermoplastic materials like poly(lactic-co-glycolic acid) (PLGA) and poly-caprolactone (PCL) possess excellent biocompatibility, but due to their high melting temperature are not suitable for bioprinting. In some cases, they are used to provide mechanical strength to hydrogel-based constructs [[21], [22], [23], [24], [25], [26]]. For example, reinforcement of GelMA hydrogels with melt-electrospin PCL fibers yielded to a significant increase in stiffness of the bioconstruct [22]. Other approaches include hybrid printing where fused PCL filaments are extruded with hydrogel bioinks to obtain bioconstructs with increased mechanical properties [21,25,[27], [28], [29]].

Bioinks containing minerals such as calcium phosphate (CaP), tri-calcium phosphate (TCP) and hydroxyapatite (HA) are frequently used due to their biocompatible and osteoinductive properties [30]. Alternative strategies for the design of bioinks have been developed to improve the shape fidelity of the bioprinted constructs. Some recent research has focused at obtaining a more accurate control of the crosslinking reaction by adding complex chemistries at the crosslinked hydrogel or by incorporating nanoscale elements to tune the rheology of the bioink [6]. Special interest has been shown for nanocomposite hydrogels such as synthetic nanosilicate clay or Laponite (LAP), due to their ability to self-assemble to form reversible, shear thinning gels. LAP is an attractive rheology enhancer and has been studied for its ability to promote cell differentiation *in vitro* and *in vivo* [[31], [32], [33]]. When combined with GelMA, LAP has showed to improve significantly the mechanical stability and biological functionality of bioprinted constructs [34]. Recently, the association of LAP with several polymeric materials to produce viable bioinks for cell printing applications has been studied [[35], [36], [37], [38]]. More specifically, LAP combined with polyethylene glycol (PEG) and alginate improved rheological properties and printability which lead to an enhancing recovery after shear [37,38].

The choice of the appropriate cell type represents another crucial issue for a functional bioprinted construct. Cells for bioprinting should be able to proliferate maintaining their own phenotype and guiding the new tissue formation. Current options from bioprinting cells include: (i) cells embedded into a hydrogel that is often loaded with bioactive molecules and growth factors to aid cell metabolism, (ii) cells individually encapsulated in microcarriers or (iii) cell aggregates (spheroids) which are deposited using extrusion printers and are allowed to self-assemble into the desired 3-D structure [7,8,[39], [40], [41], [42]].

MSCs represent the most suitable cell source for tissue regeneration approaches like bioprinting for several reasons. Firstly, they have unique self-renewal ability and the capacity to differentiate into a variety of cell types, such as osteoblasts, adipocytes, chondrocytes, tenocytes and skeletal myocytes. In addition, they possess immunomodulatory properties, they have shown the capacity to secrete protective biological factors and they can be easily purified from different tissues (such as bone marrow, adipose tissue or umbilical cord). Moreover, their therapeutic effect has been thoroughly explored in a number of human clinical trials [7,43], their use is considered safe, and ethical concerns present for embryonic and pluripotent stem cells do not apply for MSCs. On the other hand, the bioprinting of terminally differentiated cell lines like osteoblasts and chondrocytes has been explored, but their limited lifespan and the invasive surgical procedure needed for harvesting them represent the major limitations for their use [44].

Beyond cell-laden scaffolds characteristics, parameters such as the printing resolution, placement accuracy, amount of printed layers, dimension and consumed printing time should be taken into consideration during the 3D bioprinting process [30]. Common methods used to produce 3D bioproducts include (i) inkjet, (ii) laser and (iii) extrusion-based bioprinting [7,9,25,30,45].

(i) Inkjet-based bioprinting uses thermal, piezoelectric or electromagnetic forces to deposit small bioink droplets from a print head nozzle. This method is widely employed because it is easy to use, low cost and highly versatile. It allows printing many materials with high resolution and speed [7,46]. Nonetheless, tissue fabrication using this method presents limitations such as frequent nozzle clogging, thermal and mechanical stress on cells, poor cell density and non-uniform droplet size.
(ii) Laser bioprinting is a scaffold-free technique able to print cells in a very high density using laser energy to transfer and encapsulate them from a donor slide within droplets of biomaterial toward a collector slide [47,48]. Despite the high printing accuracy and 3D structure resolution of this method, cell survival is low and the printing process is expensive and time consuming. In this context, stereolithography or digital light processing represent reliable alternatives to overcome these limitations. These techniques employ ultraviolet or visible light to solidify a liquid with photopolymeric properties to layer-by-layer manufacturing the 3D construct [49,50]. However, further technical developments of laser-based techniques are needed in order to achieve large and more complex tissue reconstruction.
(iii) Extrusion-based bioprinting employs different nozzles to extrude the bioink driven by pneumatic or mechanical pressure. It is the most commonly used technique nowadays due to its very high resolution and the possibility to print high viscosity bioinks such as complex polymers and cell spheroids [45,[51], [52], [53]]. One of its major disadvantages is associated with shear-stress during printing, which may affect cell viability [54].

Recently, some interesting in-house technologies have been developed to improve the final size and structural integrity of the biological constructs and overcome the limits of the aforementioned bioprinting methods. For instance, systems such as MtoBS by Shim et al. and ITOP by Kang et al., have been recently developed to generate hybrid hydrogels, with large size and structural integrity suitable for surgical implantation [25,55]. In addition, O'Connell et al. produced a bioprinting device enabling the *in situ* 3D bioprinting of the tissue-construct for intra-operative applications [56]. The technological aspects and applications of these novel systems will be described with more details in the next sections. In addition, volumetric printing technologies have emerged recently, enabling the creation of entire objects at once in a short time (i.e. several seconds), rather than using layer-by-layer additive manufacturing. A centimeter-scale cell-laden hydrogel has been generated using this technique at an unprecedented printing velocity [57]. However, this technology is still in its infancy and further studies are needed for upscaling the production of hydrogel-based constructs and to evaluate their biological activity *in vivo*.

Alongside the development of bioink design and printing methods towards high-shape fidelity bioprinting approaches, the effective maturation of the bioprinted construct into a functional tissue analogue is still under investigation. In fact, cells actively drive the morphogenesis through their response to a combination of mechanical, biomechanical and geometrical properties. In this regard, bioreactors could be useful to provide a tissue-specific physiological *in vitro* environment during tissue maturation for tissue engineering applications. In particular, bioreactors can provide the 3D-cell culture system with a fluid dynamic microenvironment. This microenvironment facilitates a uniform distribution of nutrients and gaseous exchange, promoting intra- and intercellular interactions with the ECM and cell differentiation [26,[58], [59], [60], [61]].

## 4 Advances in bone repair

Bone defects and injuries resulting from aging, trauma, infection, disease or failed arthroplasty often require tissue reconstruction using a graft or metal implants [62]. However, these techniques show often limited efficacy due to several reasons, such as scarce bone substitute availability, donor site morbidity, poor tissue integration, fatigue fractures, immune response activation or infections [[63], [64], [65]]. Over the past decades, extensive attention has been given to 3D-printed scaffolds for bone tissue regeneration due to their 3D structure with desirable porosity and mechanical properties that can mimic the natural trabecular bone. However, current tissue engineering applications still lack the ability to organise cells within a 3D scaffold and to reproduce the microstructure of native tissues. Bioprinting is expected to be a powerful tool for bone tissue engineering, since it can build 3D constructs to reproduce the bone microstructure. Moreover, bioprinting has the potential to enhance bone repair clinical outcomes since it could overcome some of current bone graft side adverse effects.

An overview of recent *in vitro* and *in vivo* studies using 3D bioprinting for bone tissue engineering applications is shown in Table 1. Moreover, we also included some studies on 3D printing since the wide application of additive manufacturing techniques for bone tissue engineering. More specifically, reinforcing polymers such as thermoplastics as biomaterials have been extensively used as biomaterial inks in this field. Considerable work has been done to develop functional models of bone tissues in laboratories. For instance, some authors have used composite-hydrogel printed MSCs using laser-based bioprinting to treat *in situ* cranial defects in mouse [71]. Keriquel et al. use a composite-hydrogel characterised by two phases to resemble the bone ultra-structure and its mechanical resistance and osteoconductivity properties. The phase of collagen type I mimics the organic part of the bone and a second phase of nano-hydroxyapatite represents the mineral content [71]. After 42 days post-implantation, the research group showed cell proliferation and good effects in enhancing bone regeneration. Other groups have explored the use of a composite of synthetic polymeric membrane (PCL/PLGA/TCP), hydroxyapatite and growth factors (such as platelet rich plasma (PRP) and bone morphogenetic protein-2 (BMP-2)) to elicit cell differentiation and bone tissue formation *in vitro* and *in vivo* [66,73,74,76,[78], [79], [80], [81], [82], [83]]. Moreover, pre-vascularization strategies aiming to resemble the highly vascularized nature of bone have been also developed using vascular endothelial growth factors (VEGF) and endothelial cells (ECs) [41,70,72,84]. For instance, Anada et al. used GelMA hydrogel as a matrix with calcium phosphate materials (peripheral octacalcium phosphate, OCP) in which MSCs-ECs were co-cultured to drive osteogenic differentiation and blood vessel-like structures formation. In particular, the research group produced a biomimetic dual ring structure using stereolithography bioprinting technique for the precise positioning of cells and OCP material into GelMA hydrogels, showing good results in term of capillary-like structure formation already after 1 day of culture [70]. Recently, the ability of a nanoclay-based bioink to drive osteogenic differentiation of human BMSC was investigated by Cidonio et al. The bioink consisted of a blend of LAP combined with alginate and methylcellulose components. Results obtained demonstrated the capacity to obtain bone mineralized matrix *in vivo*, even in the absence of endogenous BMP-2 stimuli [31]. In the same study, the authors showed that 3D printed scaffolds alone or combined with VEGF or ECs allowed to elicit a vascular remodelling response in an *ex vivo* chorioallantoic (CAM) model. The fabrication of a suitable 3D construct for the regeneration of the osteochondral bone, which is present in joints regions such as the knee, is particularly challenging. For these applications, the different hierarchical and organizational structure of this tissue, which is composed by abundant articular cartilage and subchondral bone region, has to be mimicked. With this purpose, Shim et al. developed a multi-head tissue/organ building system (MtoBS), which is an extrusion-based system able to dispense biomaterials, including thermoplastic biodegradable materials and hydrogel, to produce 3D tissues or organs with completely different rheology properties and different cell types [55]. Using this approach, Shim et al. fabricated a PCL framework to confer structural strength to the construct, which was covered with two different cell-laden hydrogels. The obtained 3D bioprinted construct contained two different cell types (osteoblasts and chondrocytes) to resemble the osteochondral nature of the bone. Both cell types not only retained their initial position and viability, but also proliferated up to 7 days after being dispensed. More recently, Kang et al. developed another multi cartridge system, the integrated tissue organ printer (ITOP), to produce multiple tissue constructs of any shape with clinically relevant size and structural integrity. Authors reported high cell viability and density *in vitro* and a newly formed vascular network throughout the entire implantation in mice, showing the potential of their system to treat mandible and calvarian bone defects *in vitro* and *in vivo* [25]. Kang et al. demonstrated the capabilities of the ITOP system by fabricating mandible and calvarial bone, cartilage and skeletal muscle.

**Table 1:** Recent *in vitro* and *in vivo* studies using 3D bioprinting for bone tissue engineering applications.

Biomaterials and GFs	Cells	Main results	References
**Extrusion-based bioprinting**			
Alginate/gelatin/bioglass	Osteoblasts	Increased cell proliferation and mineralisation by adding bioglass to the hydrogel.	*Wang* [76]
Alginate/gelatin/HA	ASCs	Structure integrity maintained for 28 days in culture with increased storage moduli. Intense matrix formation and upregulation of osteogenic markers.	*Wenz* [77]
Alginate/GelMA/HA	BMSCs	Long-term structural integrity and high cell viability after 3 days of *in vitro* culture.	*Wüst* [74]
Alginate/gelatine/polyP-Ca^2+^ complex	Osteoblasts	Improved cell proliferation, Young's modulus and significant matrix deposition due to the calcium salt from polyP- Ca^2+^ complex.	*Neufurth.* [75]
LAP/alginate/methylcellulose ​+ ​VEGF or BMP-2	BMSCs, HUVECs	Viable and functional constructs *in vitro* (BMSCs) and *ex vivo* (HUVECs ​+ ​VEGF). Extensive mineralisation produced by BMP-2 absorbed scaffolds compared to alginate controls and BMSC-laden scaffolds after 4 weeks, supporting bone tissue formation.	*Cidonio* [31]
Alginate/PCL	Pre-osteoblasts, Chondrocytes	Maintained their cell viability and proliferation in culture of encapsulated cells dispensed into the pores of a pre-formed PCL framework.	*Shim* [55]
Bioglass/gliadin/PCL	Pre-osteoblasts	Scaffold with controllable architecture, high compressive strength, proper degradability and biocompatible. Osteogenesis *in vivo* after implantation in rabbit femoral bone defect.	*Zhang* [69]
Chitosan, Chitosan/HA, Alginate, Alginate/HA	Osteoblasts	The combination of Chitosan/HA showed viscoelastic properties, improved cell viability, proliferation and differentiation.	*Dermitaş* [78]
Collagen/PCL-HA/TCP ​+ ​BMP-2 or PRP	Osteoblasts	The addition of PRP showed higher cellular activities and mineralisation for bone tissue regeneration in Collagen/PCL-HA/TCP biocomposites, compared to the addition of BMP-2.	*Kim* [66]
Gelatin/silicate nanoparticles ​+ ​VEGF supplementation	HUVECs and BMSCs	Constructs with high structural stability, cell survival and proliferation during maturation *in vitro*.	*Byambaa* [68]
GelMA/Pluronic F-127	BMSCs	Total bone formation, increased vascularization and implant remodelling observed after implantation in rat femoral bone defect.	*Daly* [41]
Matrigel	ECs and BMSCs	Changing fiber spacing or angle of fiber deposition yielded to scaffolds of different porosity and elastic modulus. Bone and vessel formation in printed grafts after subcutaneous implantation in immunodeficient mice.	*Fedorovich* [80]
PCL/β-TCP	Fibroblasts, Pre-osteoblasts	Excellent cell affinity and mechanical properties of the membrane. Enhancement of bone formation after implantation in alveolar bone defect in beagles.	*Shim* [67]
PCL/CB(6)/DAH-HA ​+ TGFβ/Atelocollagen ​+ ​BMP-2	hTMSCs	Cytocompatible multi-layered 3D construct capable of inducing cell differentiation *in vitro*. *In vitro* and *in vivo* osteochondral tissue formation.	*Shim* [73]
PCL/TCP/Pluronic F-127	hAFMSCs	Formation of new vascularized bone tissue with no necrosis, after 5 months of calvarian bone reconstruction *in vivo*.	*Kang* [25]
**Stereolithography**			
GelMa/OCP	HUVECs and BMSCs	Bone-like tissue and capillary-like structure formation after 1 day of *in vitro* culture.	*Anada* [70]
**Laser Bioprinting**			
HA-collagen	BMSCs	Different cell printing geometries impact on bone tissue regeneration. Proliferation of printed MSCs and improved bone regeneration 42 days post-implantation in the calvarian bone defect mouse model.	*Keriquel* [47]

Abbreviations: [HA] hydroxyapatite, [ASCs] adipose derived mesenchymal stem cells, [GelMa] gelatin methacrylamide, [BMSCs] bone marrow-derived mesenchymal stem cells, [polyP-Ca^2+^ complex] calcium salt of polyphosphate-calcium complex, [VEGF] vascular endothelial growth factor, [HUVECs] Human umbilical vein endothelial cells, [BMP-2] bone morphogenetic protein-2, [PCL] poly(caprolactone), [PRP] platelet rich plasma, [TCP] tricalcium phosphates, [ECs] endothelial cells, [CB(6)] Cucurbit [6]uril, [DAH-HA] 1,6-diaminohexane-conjugated hyaluronic acid, [hTMSCs] human nasal inferior turbinate tissue-derived mesenchymal stromal cells, [TGFβ] transforming growth factor, [hAFMSCs] human Amniotic fluid-derived mesenchymal stem cells, [OCP] peripheral octacalcium phosphate.

## 5 Advances in cartilage repair

Cartilage is a dense connective avascular tissue with limited self-repair ability, which is frequently damaged as a result of trauma and degenerative joint diseases such as osteoarthritis. Osteoarthritis is characterized by a progressive loss of articular cartilage and causes pain, impaired function, limited range of motion, stiffness, catching, locking and joint enlargement or swelling [85]. In the last decades, the poor outcome of standard surgical joint replacements has triggered the development of alternative approaches including cell-based therapies and tissue engineering. Autologous chondrocyte implantation (ACI) was the first cell-based approach used to treat cartilage defects, but it presented drawbacks such as limited availability and lifespan of chondrocytes. Further disadvantages include the high cost and length of the procedure and patient discomfort related to surgery [[86], [87], [88]]. Concurrently, the intra-articular injection of MSCs has gained popularity as emerging regime for cartilage regeneration. The safety, feasibility and efficacy of MSCs for regeneration of human articular cartilage has been investigated in more of 50 clinical trials to date [89].

Cartilage repair with a personalised engineered tissue resembling the native cartilage directly into the site of lesion is a very attractive approach. However, it remains a significant challenge due to cartilage complex zonal organization, which plays an important role in the structure and function of the tissue. Some recent studies based on bioprinting were conducted to obtain the ideal graft, which would be able to integrate in the host tissue and to closely mimic the native cartilage. Moreover, the graft should maintain cartilage zonal organization, such as hyaline cartilage of articulating surface of bones or fibrocartilage of meniscus, as well as its ECM composition and mechanical properties.

An overview of recent studies using different biomaterials and cells for 3D bioprinting of cartilage is presented in Table 2. The choice of the proper cell type and biomaterial is crucial to achieve cartilage-like tissue formation. In fact, it has been demonstrated that natural hydrogels such as agarose, alginate and gelatin-methacryloyl (GelMa) drive differently MSCs phenotype and differentiation after 28 days *in vitro* culture [21]. MSCs cultured with alginate and agarose hydrogels showed higher cell viability and hyaline-like cartilage formation with predominant expression of collagen type II. When culturing MSCs in GelMA bioconstructs, a fibrocartilage-like tissue formation was observed, with higher expression of collagen type I and II. Some studies were performed to generate a human scale tissue constructs with complex architectures and high structural integrity. Kang et al. demonstrated to develop a complex, human ear-shaped construct containing cartilage tissue with histological and mechanical characteristics of human auricles after maturation *in vivo* [25]. They used a ITOP technology for the delivery of bioink, which consisted of different hydrogels (gelatin, fibrinogen and HA) and rabbit ear chondrocytes. More recently, a PCL microchamber system has been proposed to engineer scaled-up tissues with native-like collagen anisotropies. Using the PCL microchamber system, it has been possible to support the formation of orientated arrays of MSCs aggregates to produce cartilage tissues with a collagen network organization mimicking the native tissue, with a parallel orientation in the superficial zone and random-perpendicular organization in the middle and deep zones [26].

**Table 2:** Recent *in vitro* and *in vivo* studies using 3D bioprinting for cartilage tissue engineering applications.

Biomaterials and GFs	Cells	Main results	References
**Extrusion-based bioprinting**			
Agarose, Alginate, GelMA and PEGMA ​+ ​TGFβ-3	BMSCs	High levels of MSC viability observed post-printing in all bioinks. Alginate and agarose hydrogels supported the development of hyaline-like cartilage phenotype. GelMA and PEGMA-based hydrogel supported the development of fibrocartilage-like tissue. PCL microfibers increased the compressive moduli of the bioink (544 fold increase for alginate, 45 fold for GelMA). Obtained values were comparable to articular cartilage.	*Daly* [21]
COL type II hydrogel	Chondrocytes	Stable cell distribution patterns throughout the culture period with formation of new ECM with gradient distribution.	*Ren* [92]
dECM/PCL	hTMSCs	High cell viability and significant chondrogenic differentiation *in vitro*.	*Pati* [17]
GelMA	ACPCs, BMSCs and Chondrocytes	Neo-cartilage synthesis in layered co-cultures in a zonal-like architecture *in vitro*. Higher elastic modulus of the hydrogel correlates with higher cartilage matrix synthesis.	*Levato* [94]
GelMA/HAMA	IFP-MSCs	Rapid generation of Core/Shell GelMa/HAMA bioscaffolds with high compressive modulus and cell viability.	*Duchi* [96]
GelMA/HAMA	IFP-MSCs	Intraoperative bioprinting using the ‘biopen’ to treat chondral defect in sheeps showed better macroscopic and microscopic cartilage characteristics.	*Di Bella* [93]
GelMA/HAMA/CSMA	Chondrocytes	The addition of HAMA and CSMA to GelMA constructs resulted in more rounded cell morphologies, enhanced chondrogenesis, ECM production and increased compressive moduli.	*Levett* [13]
GelMA-Tyr	Chondrocytes	Neo-cartilage formation *in vitro.* Better integration *in vivo* with no damage of the surrounding tissue after *in situ* crosslinking with visible light.	*Lim* [97]
PCL/Alginate ​+ ​TGFβ3	Chondrocytes	Enhanced cartilage tissue and type II collagen fibril formation after four weeks of implantation in nude mice.	*Kundu* [27]
PCL/Pluronic F-127	Chondrocytes	High cell viability, new cartilage tissue formation and increase of GAG content *in vivo* of human ear–shaped cartilage constructs.	*Kang* [25]
SA, SA/COL, SA/AG	Chondrocytes	SA/COL showed better compressive strength, cell adhesion, proliferation and cartilage-specific gene expression. SA/COL also suppressed the de-differentiation of chondrocytes and preserved their phenotype.	*Yang* [95]
**Stereolitography**			
GelMA, HAMA	Chondrocytes	Both materials supported cartilage ECM formation and recovery of chondrocyte phenotype *in vitro*. Influence of cell density on the differentiation pattern.	*Lam* [90]
**Inkjet-based bioprinting**			
PCL microchambers	BMSCs and Chondrocytes	PCL microchambers promoted growth and fusion of cellular spheroids. Formation of stratified cartilage formation with collagen fibre architecture, composition and biomechanical properties comparable to the native tissue.	*Daly* [26]

Abbreviations: [GelMa] Gelatin methacrylamide, [PEGMA] Poly(ethylene glycol) methacrylate, [TGFβ-3] Transforming growth factor-3, [BMSCs] Bone marrow-derived mesenchymal stem cells, [COL] collagen, [dECM] decellularized extracellular matrix, [PCL] poly(caprolactone), [hTMSCs] human nasal inferior turbinate tissue-derived mesenchymal stromal cells, [ACPCs] Cartilage-resident chondroprogenitor cells, [HAMA] hyaluronic acid methacrylate, [CSMA] chondroitin sulfate methacrylate, [IFP-MSCs] infrapatellar fat pad derived mesenchymal stem cells, [GelMA-Tyr] Gelatin methacrylamide-tyramine, [SA] Sodium Alginate, [AG] agarose.

Another key challenge for bioprinting is allowing the repair of cartilage defects *in situ.* Current research efforts in the field are focus at retaining the cell-laden hydrogels at the target site and achieving a successful integration of the construct within the native tissue. For instance, poly(ethylene glycol) dimethacrylate (PEGDMA) hydrogels containing chondrocytes have been directly ink-jet printed into the cartilage defect of an osteochondral plug model *ex vivo* [91]. The *in situ* printed construct showed good chondrocytes viability and mechanical properties similar to native human articular cartilage, with cartilage ECM protein expression as collagen type II, aggrecan and glycosaminoglycans (GAGs) [91]. Similarly, a hand-held device, called the Biopen, has been developed for the simultaneous delivery of MSCs and GelMA directly into the defect site in a single session surgery. In particular, this system allows the reconstruction of different tissues by printing multiple layers using different biomaterials and/or cells, simply by changing cartridges. The Biopen integrates in one single component bioink chambers, a multi-inkjet extruder nozzle, a light source to catalyze phase transformation of the ink and a motorized extrusion system [56]. Moreover, the internal structure of the nozzle was designed to contain the bioink comprising living cells in an inner core, which is protected by a shell of a more robust biomaterial. The *in vivo* application of the Biopen was investigated by Di Bella and colleagues to treat *in situ* chondral defects in sheeps [93]. The bioink ‘core’ was filled with allogeneic infrapatellar adipose-derived stem cells (ASCs) and HA-GelMA hydrogel (hyaluronic acid and gelatin methacrylamide), which was surrounded by an outer shell of HA-GelMA bioink and the photoinitiator. The results of this pilot study show that real-time, safe, *in vivo* bioprinting is a feasible to regenerate articular cartilage in a large animal model. Moreover, better overall macroscopic and microscopic cartilage characteristics were observed with Biopen when compared to pre-constructed 3D bioscaffolds, microfractures and untreated controls at the time points studied. Lately, a visible-light responsive gelatin ink has been developed by Lim and coworkers for the *in situ* repair of cartilage defects [97]. *In situ* crosslinking using visible light showed no damage to the surrounding tissue, in contrast to UV light commonly used in biofabrication. Moreover, this gelatin bioink functionalised with tyramine and methacryloyl (GelMA-Tyr) proved enhanced adhesive strength, improved stability *in vivo* and better integration of the engineered tissue construct to the surrounding native cartilage, demonstrating its potential for intraoperative bioprinting applications.

## 6 Regulatory framework and available standards for 3D bioprinted products

### 6.1 Classification of 3D bioprinted products under EU regulatory framework

Recent discussions in the International Coalition of Medicines Regulatory Authorities (ICMRA) recognised 3D bioprinting as a particularly disruptive technology for regulatory systems [98]. An in-depth analysis showing how the current European legislative framework may be affected by the emergence of 3D bioprinting for medical purposes was released by the European Parlamentary Research Service in 2018 [99]. The main regulatory challenges identified in this analysis were defining and categorising the products and processes in 3D bioprinting; and therefore, identifying the relevant regulations that would be applicable throughout the whole process. The appropriate product category designation would also determine the necessary marketing authorization procedures and the liability regime for manufacturers in case of defective products.

A 3D bioprinted product could potentially be a medical device or an accessory to a medical device, an Advanced Therapy Medicinal Product or a medicinal product. The European Medicines Agency (EMA) has issued a scientific recommendation on the classification of viable cells cultured within a 3D structure that is part of the finished product. In that particular case, it was considered that the product would fall within the definition of a tissue engineered product, combined Advanced Therapy Medicinal Product (ATMP) [100]. In accordance with this recommendation, a 3D bioprinted meniscus composed of a scaffold seeded with different cells derived from the patient pluripotent stem cells would be likely classified also as a combined ATMP [101] in Europe; but the scaffold should also demonstrate compliance with the applicable regulation for medical devices (MDR) [102].

### 6.2 EMA's approach to regulating combined ATMPs

ATMPs are a special class of medicines (including gene therapies, somatic cell therapies and tissue engineered products) that are governed by Regulation 1394/2007 in Europe [101]. Despite the fact that nowadays it is common to have combinations of medicines and devices, there is no available classification list of “combination ATMP products”. Therefore, each classification is performed by EMA on a case-by-case basis and the distinction between combined or non-combined ATMPs is often subject to discussion during classification procedures [103].

The whole product considered a combined ATMP shall be subject to final evaluation by EMA via the centralised procedure for premarket approval, as stated in Article 9 of the ATMP Regulation. The Committee for Advanced Therapies (CAT) is responsible for assessing the quality, safety and efficacy of ATMPs. The CAT prepares a draft opinion on each ATMP application submitted to EMA, before the Committee for Medicinal Products for Human Use adopts a final opinion on the marketing authorisation of the product concerned [101]. EMA also manages the coordination of the consultation with the Notified Body in charge of the conformity assessment of the medical device, and should recognise the results of the assessment of the medical device [104,105]. The specific requirements for the authorization of ATMPs-containing devices are listed in Article 7 of the ATMP Regulation: “*For ATMPs containing medical devices, biomaterials, scaffolds or matrices, a description of the physical characteristics and performance of the product and a description of the product design methods, in accordance with Annex I to Directive 2001/*83/EC.” [101].

It is important to note that the medical device should retain its original form and function to be considered as an “integral part” of the final product to classify it as a combined ATMP. For instance, the CAT committee considered that pancreatic beta cells embedded in an alginate matrix for the treatment of diabetes were somatic cell therapy medicinal products, and not combined ATMPs. This was because the alginate matrix was reworked by the cells during culture to support the biological features and functional activities of the cells and the matrix function was no longer considered to be linked to its structural properties [103]. Therefore, by analogy, it could be possible that a 3D bioprinted product would not be considered a combined ATMP but a tissue-engineered product.

### 6.3 Other applicable regulations to the 3D bioprinting process

**Fig. 3:**
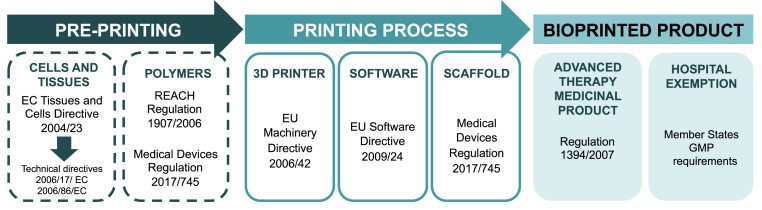
Scheme of the regulatory framework applicable in Europe for the different steps of the bioprinting process and the final product. Good manufacturing practice (GMP).

Furthermore, other legislations are relevant at different steps of the production of 3D bioprinted products. We have gone through the whole 3D bioprinting process and identified the applicable legislations in the following steps: (i) pre-printing, (ii) printing process and (iii) final bioprinted product (see Fig. 3 attached).

#### 6.3.1 Pre-printing

• Cells are the core element of the bioprinting process, and the legal framework defining the safety and quality standards for tissues and cells is set out in the European Union Tissue and Cells Directive (Directive 2004/23/EC) [106]. This Directive is applied through two separate implementing Directives: the First Technical Directive, covering the steps of donation, procurement, and testing of tissue and cells and the Second Technical Directive covering their storage and distribution [107]. When appropriate, additional requirements from ATPMs Regulation 1394/2007 should be included.
• The REACH Regulation 1907/2006 for the Registration, Evaluation, Authorisation and Restriction of Chemicals and Regulation 1272/2008 on classification, labelling and packaging of substances and mixtures [108] apply to all chemical substances, such as natural or synthetic polymers used for cell culture or in the bioink. Moreover, the polymeric matrix in which cells are cultured, can also fall under the provisions of the Medical Devices Regulation [102].

#### 6.3.2 Printing process

• The 3D printer itself would be considered as an advanced manufactured technology, which would fall under the scope of the EU Machinery Directive 2006/42 with its own quality and safety requirements [109].
• With regard to the 3D printing software, a distinction must be made according to the intended purpose. When a software is intended to be used for therapeutic or diagnostic purposes (for instance, preoperative or surgical planning), it would qualify as a stand-alone medical device under the new EU MDR [102]. In that case, the software is subject to safety and performance requirements, and would fall into the same category as the device. However, 3D design software is not classified as a medical device since it is only used for the production of the device and does not have a specific medical purpose. Therefore, the EU Software Directive 2009/24 would apply.
• If present, the scaffold used for bioprinting would constitute a medical device in accordance with Article 2 of MDR and it would need to be compliant with the medical devices regulation [102]. In the new MDR there is a clarification on mass-produced products that need to be adapted to a patient that changes the concept of custom-made products, custom-made devices are created only once from scratch following the prescription of the practitioner [102]. For instance, a 3D-printed implant that is printed with the manufacturer's standard procedure and that afterwards is adapted to the patient following the indications of the practitioner would not be anymore considered as a custom-made device but a mass-produced product. Therefore, a conformity assessment procedure needs to be undertaken for that scaffold in order to get a CE mark for that intended use by a Notified Body. Custom-made devices need to be compliant with less constraining requirements such as the general obligations of manufacturers described in Article 10 and General Safety and Performance Requirements in Annex I. These obligations include conformity assessment procedures in order to fulfill safety and performance requirements but do not include CE marking.

#### 6.3.3 Final bioprinted product

Lastly, as already described before, the final 3D bioprinted product has to be approved; most probably by EMA following the centralised route for ATMPs. However, in order to facilitate access of patients to new treatments for unmet medical needs, the ATMP Regulation includes a “hospital exemption” for products not intended to be marketed [110]. This provision is made for ATMPs that are: (I) prepared on a non-routine basis, and (II) used within the same Member State in a hospital under the exclusive responsibility of a medical practitioner, and (III) comply with an individual medical prescription, (IV) for a custom-made product for an individual patient. In that case, the EU medicines legislation would not apply and a national competent authority would have to authorise the manufacturing of such products, which must comply with the same national requirements concerning good manufacturing practice and pharmacovigilance applicable to authorised medicinal products [111]. 3D bioprinted products could eventually fall under the hospital exemption, even if this should not be the main route for their commercialisation. However, the meaning of “non-routine basis” and “custom-made” are not specified in the regulation and there is a lack of a harmonised approach for the application of the exemption among different European countries [112,113]. In fact, the hospital exemption was the topic triggering most responses and with more conflicting views manifested during the public consultation of the ATMP Regulation [114].

### 6.4 Regulatory framework for 3D bioprinting outside Europe

To our knowledge, there is also a lack of specific regulatory frameworks for bioprinting products outside Europe. The US Food and Drug Administration and Health Canada have released guidance documents with recommendations for additive manufactured medical devices [115,116], but there is no specific reference to bioprinting in these guidance documents. Australia has proposed a regulatory scheme for personalised medical devices including 3D-printed devices, which has recently been under consultation [117]. This proposed reform to the regulation of medicines and medical devices specifically mentions 3D bioprinting or printing of patient specific implants that incorporate human origin material. In the particular case of medical devices that contain human origin material (either viable or non-viable) as a component (not wholly comprised of), they would not be regulated as biologicals but as Class III medical devices with a biological component. Also South Korea and Japan have provided some specific regulatory guidance applicable to 3D bioprinting [118].

### 6.5 Available standards

Another challenge for both regulators and manufacturers pertains to the limited standards that are available covering additive manufacturing and bioprinting. Discussions within the ICMRA highlighted the importance of the adoption and update of established standards to ensure the quality, comparability, stability, safety and effectiveness of bioprinted products [98]. The standardization of biomaterials, manufacturing processes and quality control systems would notably support the clinical translation of bioprinted constructs [119]. These standards would include Good Manufacturing Practice (GMP), International Council of Harmonisation standards (ICH), International Standardisation Organisation (ISO) or ASTM (American Society for Testing and Materials) International standards.

The ASTM Committee F04 on Medical and Surgical Materials and Devices has published several working items related to 3D bioprinting such as New Test Methods for Printability of Bioinks and Biomaterial Inks (WK65680), in collaboration with the Standards Coordinating Body for Gene, Cell, and Regenerative Medicines and Cell-Based Drug Discovery (SCB). This test method aims at comparing printability and help to establish reproducibility and quality control between different material lots or manufacturers. Recently, another test method has been released on Printability of Bioinks for Extrusion-based Bioprinting (ASTM WK72274). This standard is focused on two test methods to evaluate printability of bioinks made of any material, including material inks without cells, used during extrusion-based bioprinting. ASTM efforts are also addressed at the development of a guidance document providing material properties and compositions that promote survival of living cells contained bioinks (ASTM WK65681 - New Guide for Bioinks and biomaterial inks used in bioprinting). The SCB is also coordinating two standards projects on (1) bioprinter software and data governance and (2) bioprinting hardware and component specifications, to develop guidelines for the calibration, compatibility, and interoperability of bioprinter hardware and related components.

ISO and ASTM International have a coordinated effort for the development of standards in additive manufacturing and have jointly crafted the *Additive Manufacturing Standards Development Structure*. This framework structure is aimed at identifying standards-related gaps and needs, prioritise standards areas and improve usability and acceptance among the 3D printing community, including manufacturers, entrepreneurs, consumers and others. Based on this structure, standards are developed at three levels: (i) General standards (such as concepts, common requirements, guides or safety) (ii) Standards for categories of materials or processes and (iii) Standards for a specific material, process or application. In each level, there is a division for feedstock materials, process/equipment and finished parts. Although these standards are not specific for bioprinting, they could be useful for the development and implementation of a standardised methodology for the characterisation of 3D bioprinted products.

## 7 Conclusions and future perspective

3D bioprinting technology and its applications are rapidly evolving to release personalised treatments for each patient, with the potential to revolutionise healthcare. 3D bioprinting has particularly advanced in the orthopaedics field, especially in custom-made prostheses and implants for bone and cartilage tissue engineering. Many ongoing research efforts are aimed at obtaining 3D tissue-grafts of greater complexity with better ability to mimic tissue behavior *in vivo*. Hydrogel-based matrices have received considerable interest as tissue scaffolds due to their biocompatibility and structural similarities to natural ECM. The use of composite materials combining polymer ceramic or nano-materials has shown to improve biomaterial strength and biodegradability while inducing an adequate host immune response. Furthermore, there is a growing use of dECM in scaffolds, hydrogels and bioinks due to its resemblance with the native ECM. Concerning the type of cells used in the bioink, mesenchymal stem cells from bone marrow and adipose tissue are the most widely and successfully bioprinted ones. Notable success has already been achieved using extrusion-based bioprinting with alginate carriers and scaffold-free bioinks for cartilage and bone tissue bioprinting. However, there are still some limitations to overcome before this technology reaches clinical application, *e.g.* the standardisation of the bioprinting process, scaffold production and cell source selection, in order to achieve better reproducibility of the process.

Beyond the scientific and technical challenges, 3D bioprinting has been identified as a disruptive technology for traditional health regulatory systems since different aspects of 3D bioprinting could lead to regulatory oversight. Careful examination of the existing regulatory paths in Europe indicated that a 3D bioprinted product would be classified as a tissue engineered combined medicinal product that would fall under the scope of the ATMP Regulation. Other regulations would apply at different steps of the bioprinting process, but existing regulatory frameworks do not account for the differences between 3D printing and conventional manufacturing methods.

To truly facilitate the development of these innovative products while protecting patients, there is need for adequate standards to ensure that a bioprinted product would be reproducible, with high quality, effective and safe. As an emerging field with different manufacturers and researchers developing products independently, there is still a lack of bioprinting-specific standards. A detailed analysis of the suitability of existing standards from other sectors would be relevant to enlarge the portfolio of available standards for bioprinting. Such cross-fertilisation, in terms of learning from the methodologies and guidelines existing in other fields, would support a smooth and safe translation of these products to clinical applications. Moreover, a close collaboration between academia, industry and regulators will be essential to move the field forward to facilitate patient access to these new products.

## Disclaimer

The information and views set out in this study are those of the author(s) and do not necessarily reflect the official opinion of the European Commission. The European Commission does not guarantee the accuracy of the data included in this study. Neither the European Commission nor any person acting on the European Commission's behalf may be held responsible for the use that may be made of the information contained therein.

## Funding sources

This work was supported by the 10.13039/501100000900European Commission Joint Research Centre (JRC) through the JRC Work Programme for 2019–2020, running under 10.13039/100010661Horizon 2020, the current EU Framework Programme for research and innovation funding.

## Author contributions

DS: Conceptualization, Methodology, Formal analysis, Investigation, Writing - Original Draft, Writing - Review & Editing, Visualization.

PU: Conceptualization, Methodology, Formal analysis, Investigation, Writing - Original Draft, Writing - Review & Editing, Visualization.

ST: Conceptualization, Writing - review & editing.

GC: Conceptualization, Writing - Review & editing.

JB: Conceptualization, Methodology, Supervision, Project administration, Writing - Review & editing.

## Declaration of competing interest

The authors declare that they have no known competing financial interests or personal relationships that could have appeared to influence the work reported in this paper.
